# RNA-binding protein YBX1 promotes cell proliferation and invasiveness of nasopharyngeal carcinoma cells *via* binding to AURKA mRNA

**DOI:** 10.7150/jca.56262

**Published:** 2021-04-07

**Authors:** Yuanyuan Ban, Yixin Tan, Xiaoling Li, Xiayu Li, Zhaoyang Zeng, Wei Xiong, Guiyuan Li, Bo Xiang, Mei Yi

**Affiliations:** 1 Hunan Key Laboratory of Cancer Metabolism, Hunan Cancer Hospital and the Affiliated Cancer Hospital of Xiangya School of Medicine, Central South University, Changsha 410013, Hunan, China.; 2 The Key Laboratory of Carcinogenesis and Cancer Invasion of the Chinese Ministry of Education, Cancer Research Institute and School of Basic Medical Sciences, Central South University, Changsha 410078, Hunan, China.; 3 The Key Laboratory of Carcinogenesis of the Chinese Ministry of Health, Xiangya Hospital, Central South University, Changsha 410008, Hunan, China.; 4 Hunan Key Laboratory of Nonresolving Inflammation and Cancer, The Third Xiangya Hospital, Central South University, Changsha 410013, Hunan, China.; 5 Department of Dermatology, The Second Xiangya Hospital, The Central South University, Changsha 410011, Hunan, China.; 6 Department of Dermatology, Xiangya Hospital, Central South University, Changsha 410008, Hunan, China.

**Keywords:** RNA binding protein, posttranscriptional regulation, YBX1, AURKA, translation.

## Abstract

**Background:** RNA-binding proteins (RBPs) play essential roles in post-transcriptional control of gene expression. Dysregulation of RBPs is intensively implicated in development and progression of human diseases, including cancers. However, the roles of RBPs in nasopharyngeal carcinoma (NPC), which is a distinct subtype of head and neck cancer, remain elusive.

**Methods:** NPC-related RBPs were explored by analyzing GEO database and high-throughput proteomic data obtained from crosslinking immunoprecipitation. The expression levels of Y box binding protein 1 (YBX1) protein in NPC samples were measured by immunohistochemistry (IHC) staining. The association of YBX1 protein levels with prognosis of NPC patients was analyzed by Kaplan-Meier Plotter. The expression levels of YBX1 in NPC cells were inhibited by RNA interference. Cell growth was measured by CCK-8 assay. Cell mobility and invasiveness were measured by transwell assays. Tumorigenicity was measured by using a xenograft tumor assay. The expression levels of mRNAs or proteins were determined by qPCR or western blot assays, respectively. The mRNAs binding to YBX1 were determined by RNA immunoprecipitation (RIP) and qPCR. The effect of YBX1 on mRNA translation was measured by luciferase reporter assay.

**Results:** In the present study, we demonstrated a differentially expressed RBPs profile between NPC and its normal counterpart. Among these aberrantly expressed RBPs, YBX1 was overexpressed in NPC. We found that YBX1 is mainly localized in the cytoplasm of NPC cells. Loss of YBX1 led to reduced cell proliferation, migration and invasiveness *in vitro*, and reduced tumorigenicity *in vivo*. Overexpression of YBX1 associates with high expression of cell cycle G2/M checkpoint modulators. In addition, YBX1 promotes AURKA protein expression by directly binding to its mRNA. Loss of YBX1 leads to reduction of AURKA protein level. Forced expression of AURKA rescues cell proliferation and invasiveness in YBX1-silenced NPC cell.

**Conclusions:** The current study indicated that YBX1 promotes NPC cell proliferation and invasiveness through enhancing protein synthesis of AURKA.

## Introduction

Before being translated into protein, RNA undergoes various post-transcriptional processing, including nuclear export, splicing, capping, polyadenylation, and chemical modifications. These post-transcriptional regulations largely affect the fate of mRNAs and the expression levels of the encoded proteins. RNA molecules are bound by various RNA-binding proteins (RBPs). RBPs play an important regulatory role in both transcriptional and post-transcriptional processing of RNA. For example, RBPs control pre-mRNA splicing, capping, and polyadenylation, as well as mRNA localization, stability, translation and turn over 1. Dysregulation of RBPs is intensively involved in human diseases, including cancers. Growing evidence suggests that dysregulated RBPs play essential roles in tumorigenesis. For example, YBX1, a DNA/RNA binding protein containing a cold-shock domain, server as an oncoprotein that regulates cell proliferation, survival, drug resistance, and chromatin instability in human cancers 2.

Nasopharyngeal carcinoma (NPC) is a distinct subtype of head and neck cancer originated from the mucous epithelium of the nasopharynx 3-6. Certain genetic factors associate with the development of NPC 6-8. Other pathogenic factors include Epstein-Barr virus (EBV) and environmental chemical carcinogens 5, 9, 10. Despite the general significance of RBPs in cancer development, the roles of RBPs in the development and progression of NPC remain elusive.

In this study, we found that YBX1 is overexpressed in NPC tissues. High expression of YBX1 associates with unfavorable clinical outcome in head and neck cancer patients. Silencing YBX1 in NPC cells attenuates cell growth, mobility and invasiveness *in vitro*, and tumorigenicity *in vivo*. YBX1 binds directly to AURKA mRNA and enhances its protein level in NPC cells. This study suggests that YBX1 is a novel therapeutic target for NPC.

## Materials and methods

### Dataset analysis

The gene expression profile of NPC GSE12452, which contains 31 NPC and 10 normal samples, was obtained from Gene Expression Omnibus (GEO) database 11. The expression level of YBX1 in various cancers tissues and corresponding normal tissues were analyzed by Gene expression profiling interactive analysis (GEPIA)^12^ (http://gepia.cancer-pku.cn/) and GTEx data 13. The association of YBX1 expression to patient overall survival (OS) of head and neck cancer patients was analyzed by Kaplan-Meier Plotter software based on TCGA data (http://kmplot.com/analysis/index.php?p=background&tdsourcetag=s_pcqq_aiomsg).

### Tumor tissue sample and immunohistochemistry

A cohort of 39 tumor samples from NPC patients without any treatment was obtained from the Pathology Department of Cancer Hospital of Hunan Province (Hunan, P.R. China). All these NPC samples were diagnosed as undifferentiated carcinoma. Use of clinical samples was approved by the Ethics Committee of Cancer Hospital of Hunan Province (Hunan, P.R. China). IHC staining was performed by using the methods described in previously study 10. To measure the expression of YBX1 protein in NPC tissue samples, IHC staining was carried out by using rabbit polyclonal antibody against YBX1(1:200 dilution, Abcam, Cat no. ab76149). A staining index (values, 0-6) was used as previously demonstrated 10. Briefly, the immunoreactive score was calculated by the sum of staining intensity (scores: negative = 0, weak = 1, moderate = 2, or strong = 3) and percentage (%) of stained tumor cells (scores: < 10% = 1, 10%-50% = 2, > 50% = 3). Scores from 0 to 2 were recognized as low expression and scores from 3 to 6 were recognized as high expression.

### Cell lines and culture

NPC cell lines, HK1 and C666-1, were routinely maintained in RMPI-1640 medium containing 10% fetal bovine serum (FBS) (Invitrogen, Carlsbad, CA) and penicillin/streptomycin (GIBGO, Grand Island, NY, USA) in a humidified incubator at 37°C with 5% CO_2_ and 95% air. FaDu, a hypopharyngeal carcinoma cell line, was routinely maintained in our lab. YBX1 stably silenced HK1 cells were established by using shRNA expressing lentiviral system. The sequences of shRNA targeting YBX1 were listed as followed. shRNA#1: GACGGCAATGAAGAAGATAA, shRNA#2: GTTCAATGTAAGGAACGGAT.

### siRNA transfection

Transient transfection of YBX1 targeting siRNAs was performed by using Lipofectamine RNAiMAX Reagent (Invitrogen, Carlsbad, CA) according to the manufacture protocol. The sequences of siRNAs used in this study were listed as follow. siRNA#1: GACGGCAATGAAGAAGATAA, siRNA#2: GTTCAATGTAAGGAACGGAT. The cells were cultured in medium without FBS for 8 hours and then replaced with fresh medium with 10% FBS.

### Immunofluorescence staining

Immunofluorescence staining of YBX1 protein was performed by using anti-YBX1 antibody as described previously 10. Images were captured under a Laser Scanning Microscopy (UltraView, Perkin Elmer, Cambridge, UK).

### Western blotting

Cells were lysed by using RIPA lysis buffer (Solarbio, Beijing, China) containing the protease inhibitor Cocktail (Bimake, Houston, TX, USA). Western blot analysis was performed according to previous experimental procedures 14. The following antibodies were used in this study: polyclonal anti-YBX1 (Abcam, Cat no. ab76149), polyclonal anti-AURKA (Abclonal, Cat no. A2121), polyclonal anti-BUB1B (Abclonal, Cat no. A14525), and anti-GAPDH (BBI Life Sciences, Cat no. D19090-0100).

### RNA extraction and quantitative real-time PCR (RT-qPCR)

Total cellular RNA was extracted by using Trizol regent (Invitrogen, Carlsbad, CA). Complementary DNA (cDNA) was synthesized by using the reverse transcription kit (Thermo Fisher Scientific - CN) according to the manufacture protocol. RT-qPCR was performed by using 2x SYBR Green qPCR Master Mix (Bimake). The sequences (5′-3′) of primers used in this study were listed as followed.

YBX1-forward primer: ACAAGAAGGTCATCGCAACG; YBX1-reverse primer: AACTGGAACACCACCAGGAC; AURKA-forward primer: TCCATCTTCCAGGAGGACCA; AURKA-reverse primer: TCCAAGGCTCCAGAGATCCA; BUB1B-forward primer: CAGAAACAGAATCCACGATCCC; BUB1B-reverse primer: AGACCTGTAGGTGTTCCTTGA; GAPDH-forward primer: ACGGATTTGGTCGTATTGG; GAPDH-reverse primer: TTGATTTTGGAGGGATCTCG.

### Migration and invasion assays

Cell migration and invasion assays were performed by using the transwell chambers without or with Matrigel (BD Biosciences, Bedford, MA) as previously demonstrated 10. Briefly, single cell suspensions at a density of 1×10^5^/200 μL in RPMI-1640 medium without FBS were plated in the upper chamber, then the chambers were inserted into the lower chamber containing culture medium with 15% FBS. Cells were cultured for additional 24 or 36h. Then the migrated or invaded cells were fixed with 4% paraformaldehyde and stained by crystal violet. Cell numbers were counted under microscope. The average numbers of migrated or invaded cells from five random fields were calculated.

### CCK-8 assays

Cell suspensions were planted into 96-well plates at a density of 1000 cells/200μL in culture medium. Relative cell growth was measured by using Cell Counting Kit-8 (CCK-8) (bimake) at the indicated times.

### Xenograft tumor formation assays

All animal experiments were conducted in accordance with the guidelines issued by the Ethics Committee of central south university, Hunan, China. Cells were suspended in serum-free RMPI-1640 medium and then were subcutaneously injected into the inguinal mammary fat pad of 6- to 8-week-old male BALB/c nude mice. At the end of the experiments, xenograft tumor tissues were fixed in 4% saline-buffered formalin and embedded into paraffin. Paraffin embedded tissue samples were sectioned at 5 μm, and then stained with H&E.

### RNA immunoprecipitation (RIP) assay

Cells grown at 10 cm dish were scraped off and lysed with GLB buffer (Tris-HCl pH 7.510mM, NaCl 10mM, EDTA 10mM, Triton-X 0.5%, DTT 1mM, PMSF 10mM, protease inhibitor cocktail) containing RNase inhibitor for 30 min. Cell lysate was centrifuged at 13,000 g for 15 min at 4 °C. The supernatant was collected and pre-precipitated with 20μL of recombinant protein A/G agarose (GenScript, Nanjing, China) for 1 h at 4 °C to remove non-specific binding, then the supernatant was precipitated with YBX1 antibody or IgG (Temecula, 2519253). RNAs bond with protein-antibody complex were extracted by Phenol/Chloroform extraction methods. Finally, the purified RNA was reversely transcribed and detected by qPCR.

### *In vivo* translation assay

The wild type of 5′-UTR, 3′-UTR of AURKA (WT-luc-5′-UTR, WT-luc-3′-UTR) or mutant 3′-UTR lack of YBX1 binding consensus (MT-luc-3′-UTR) were sub-cloned into pMIR-Report Luciferase vector. The luciferase constructs were transfected into HEK293 cells with or without YBX1 plasmids. The pRL-TK vector was co-transfected as internal control for transfection efficiency. Cells were collected by using 1×passive lysis buffer (Promega, Madison, WI, USA) after 24 h of transfection. The activity of firefly and renilla luciferase was measured according to the manufacture protocol by Minilumat LB 9506.

### Statistical analysis

Statistical analysis was performed using GraphPad Prism software. All data were shown as mean ± standard deviation. T-test was used to compare the two groups of variables. Spearman test was used to analyze the correlation between the expression of YBX1 and AURKA or BUB1B. Difference was considered statistically significant when *P*< 0.05.

## Results

### Identification of differentially expressed RBPs in NPC

**Figure 1:**
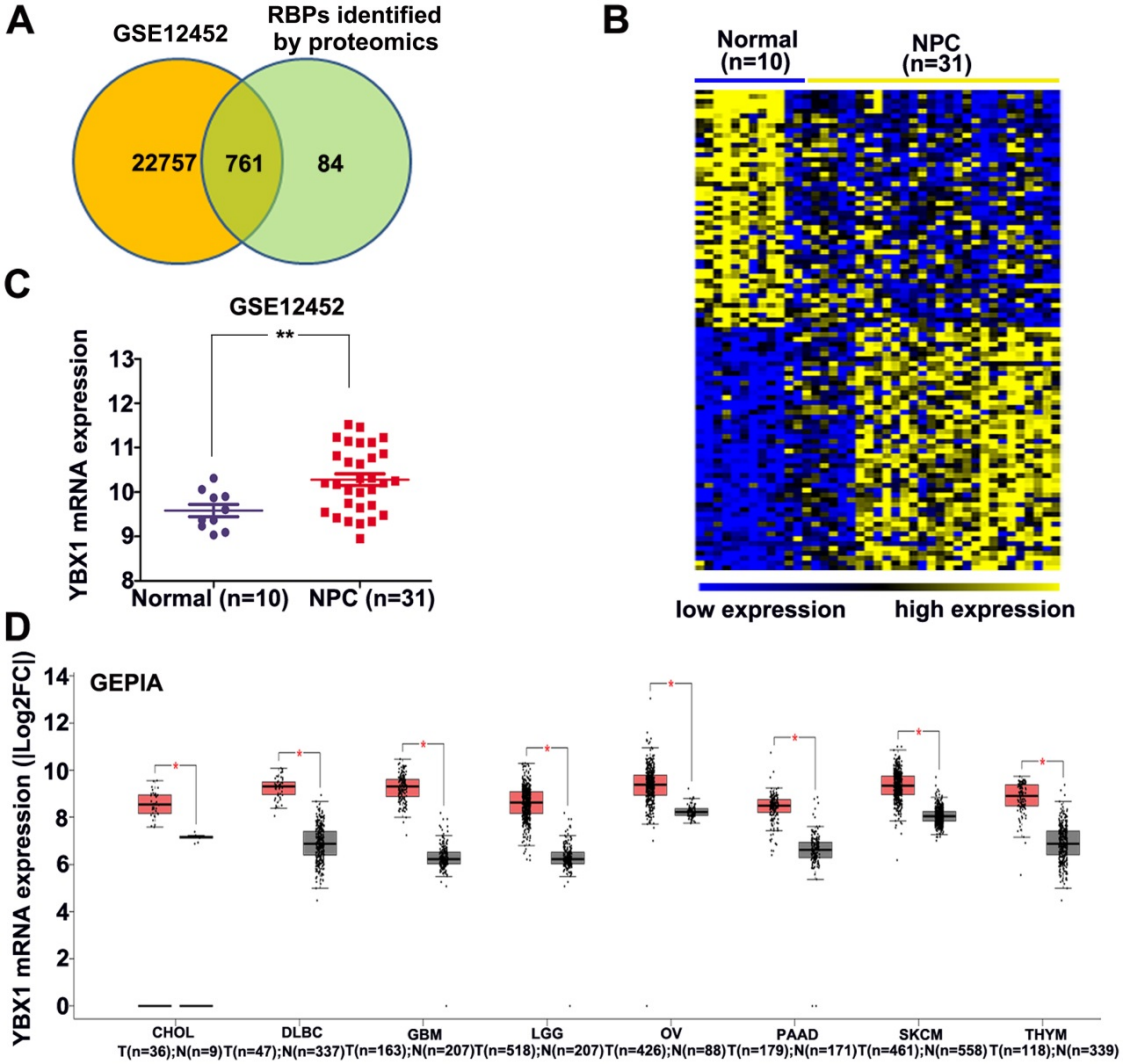
** Identification of YBX1 as an overexpressed RBP in NPC.** (A) Venn diagram depicting RBPs encoding genes detected in normal nasopharynx tissues or NPC samples. (B) Differentially expressed RBPs encoding genes between normal nasopharynx tissue and NPC were showed in heatmap. (C) The mRNA level of YBX1 was increased in NPC. (D) YBX1 was up-regulated in a variety of human cancers. *P < 0.05, **p < 0.01.

**Figure 2:**
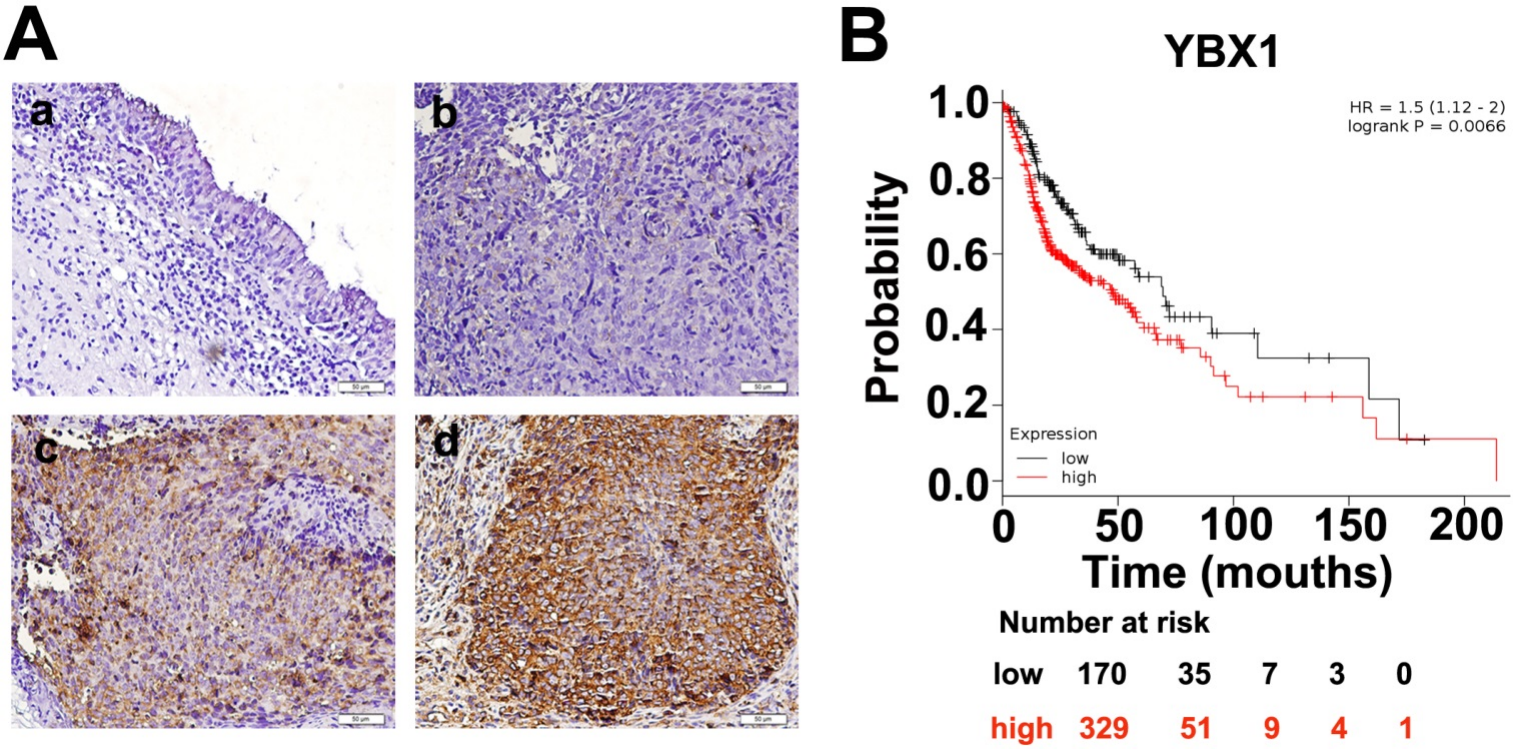
** Expressions of YBX1 protein in NPC samples.** (A) IHC staining demonstrated that YBX1 protein is overexpressed in NPC samples. *a*, negative staining of YBX1 in normal nasopharyngeal mucosa. *b*, negative staining of YBX1 in NPC. *c*, moderate staining of YBX1 in NPC. *d*, strong immunostaining of YBX1 protein in NPC. (B) High level of YBX1 mRNA predicts unfavorable overall survival in head and neck cancer patients. Data was analyzed by Kaplan-Meier Plotter Software according to TCGA dataset.

An atlas of 845 RBPs was previously identified from HeLa cells 15. We compared the expression levels of these 845 RBPs between normal nasopharynx tissue and NPC samples by analyzing dataset GSE12452. A total of 761 RBP coding genes are expressed at least either in normal or NPC samples (Figure 1A). Among these 761 RBP coding genes, 277 genes were upregulated, whereas 100 genes were downregulated in NPC as compared to normal nasopharynx tissues. The top 50 upregulated RBPs and the top 50 downregulated RBPs were displayed in heatmap (Figure 1B). Among these differentially expressed RBPs, YBX1 is highly expressed in NPC samples as compared to its normal counterparts (Figure 1C). Furthermore, YBX1 was found to be up-regulated in a variety of human cancers by analyzing GEPIA database (Figure 1D). We measured YBX1 protein level in 39 NPC samples by IHC staining. The results showed that YBX1 protein is highly expressed in NPC tissues samples but was absent in normal nasopharynx epithelium (Figure 2A). Moreover, we found that high expression of YBX1 predicts unfavorable overall survival of head and neck squamous cell carcinoma (HNSCC) patients (Figure 2B). Thus, our data suggests that YBX1 may play an indispensable role in the development of NPC.

### YBX1 promotes NPC cells proliferation and mobility *in vitro* and tumor formation *in vivo*

**Figure 3:**
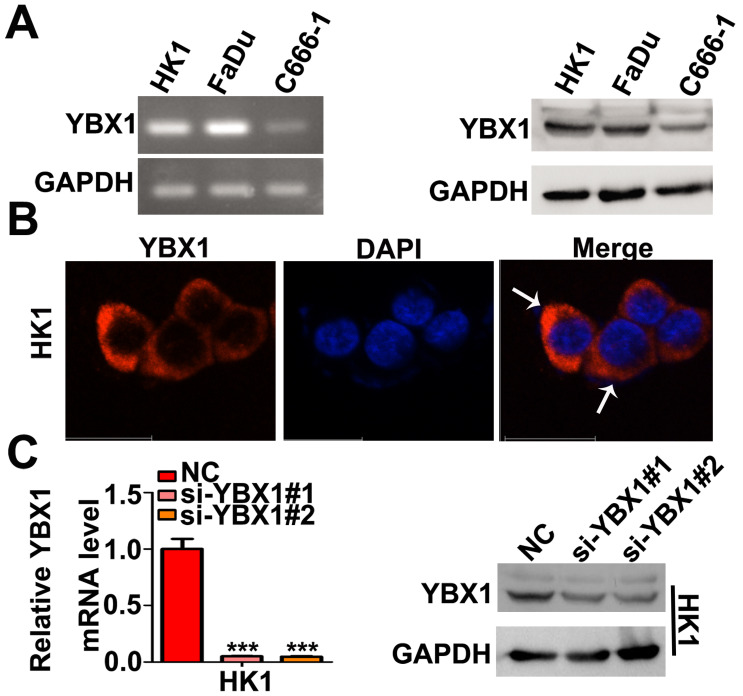
** Expression and localization of YBX1 in NPC cell lines.** (A) Expression of YBX1 mRNA and protein in HK1, FaDu and C666-1cells were analyzed by RT-PCR or western blot, respectively. (B) Immunofluorescence assay showed that YBX1 protein was primarily distributed in cytoplasm in HK1 cell. Arrow showed YBX1-expressing cells. (C) Expression levels of YBX1 mRNA and protein in mock or siRNAs transfected HK1 cells were analyzed by RT-PCR or western blot, respectively. ***p < 0.001

**Figure 4:**
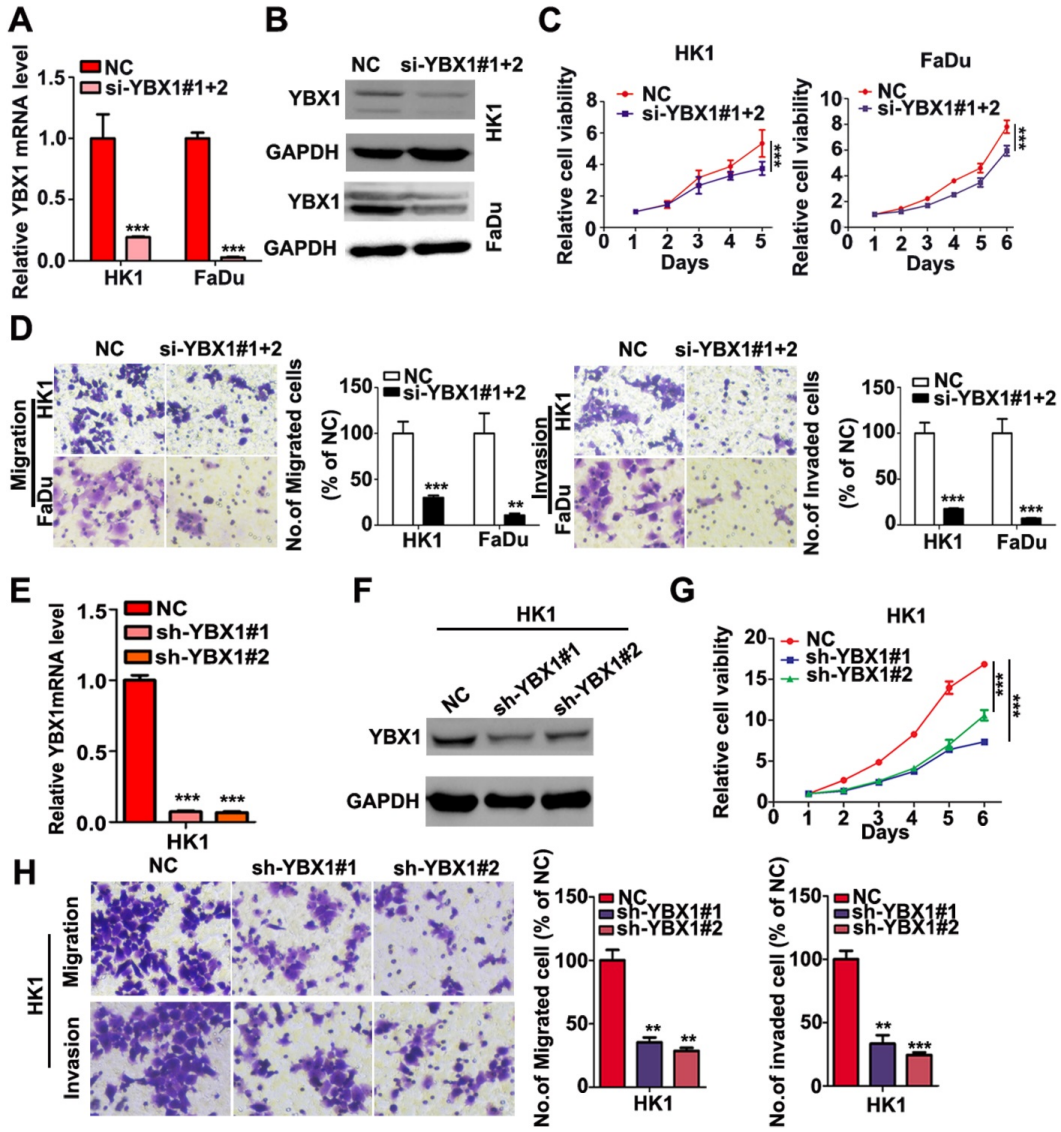
** Loss of YBX1 suppressed NPC cell proliferation, migration and invasiveness *in vitro*.** (A), (B) HK1 and FaDu cells were transfected with siRNAs mixture, then mRNA and protein levels of YBX1 were determined by qPCR or western blot assays. (C) CCK-8 assays demonstrated that loss of YBX1 suppressed cell growth of HK1 and FaDu cells. (D) Loss of YBX1 inhibited cell migration and invasion *in vitro*. (E), (F) The mRNA and protein levels of YBX1 in control or shRNAs expressing lentivirus infected HK1 cells were determined by qPCR or western blot assays. (G) CCK-8 assays demonstrated that stable silencing of YBX1 delayed cell growth of HK1 cell. (H) Stable silencing of YBX1 repressed cell migration and invasiveness of HK1 cell *in vitro*. **p < 0.01, ***p < 0.001

**Figure 5:**
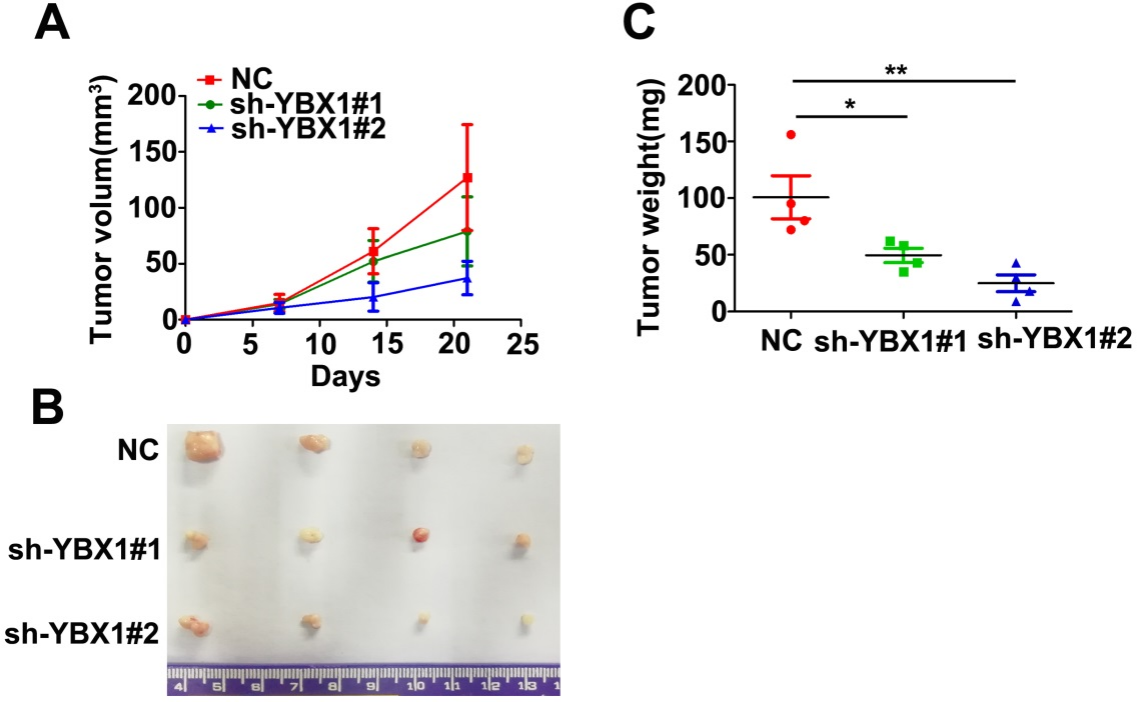
** Knockdown of YBX1 suppressed tumor formation *in vivo*.** (A) Growth curve of xenograft tumor derived from control or YBX1 silenced HK1 cells in nude mice. (B) Macroscopic view of xenograft tumors isolated from nude mice at the end point of experiment. (C) Weight of xenograft tumors derived from control or YBX1 silenced HK1 cells. *P < 0.05, **p < 0.01.

We measured the mRNA and protein levels of YBX1 in three NPC cell lines (HK1, FaDu and C666-1) by RT- PCR and western blot assays, respectively. As shown in Figure 3A, the mRNA and protein levels of YBX1 were highly expressed in HK1 and FaDu cell lines but were much lower in C666-1 cells (Figure 3A). Immunofluorescence assay showed that YBX1 protein was mainly distributed in the cytoplasm of HK1 cells (Figure 3B), suggesting that it acts primarily as a RBP in NPC, rather than a transcription factor. To further investigate the roles of YBX1 in NPC progression. We silenced YBX1 by transient transfection of targeted siRNAs (siRNA#1, siRNA#2). RT-qPCR and western blot assays showed that transfection of siRNAs efficiently reduced the mRNA and protein levels of YBX1 in HK1 cells (Figure 3C). In order to achieve high efficiency of RNA interference, the mixture of two siRNAs were co-transfected into HK1 and FaDu cells (Figure 4A&B). CCK-8 assays demonstrated that that transient silencing of YBX1 led to inhibition of cell proliferation in HK1 and FaDu cells (Figure 4C). In addition, transient silencing of YBX1 inhibited cell migration and invasion (Figure 4D). Then YBX1 was stably silenced by shRNA expressing lentivirus in HK1 cells (Figure 4E&F). Stable silence of YBX1 attenuated HK1 cells proliferation (Figure 4G), migration and invasiveness (Figure 4H). Xenograft tumor formation assays further demonstrated that loss of YBX1 in HK1 cells delayed tumor growth in nude mice (Figure 5A). As shown in Figure 5B, C&D, the weight of xenograft tumors from YBX1-silenced cells were much lowering than that of derived from control cells. Thus, our data suggested that YBX1 plays a tumor promotive role in NPC.

### High expression of YBX1 correlates with overexpression of G2/M checkpoints regulators in NPC

**Figure 6:**
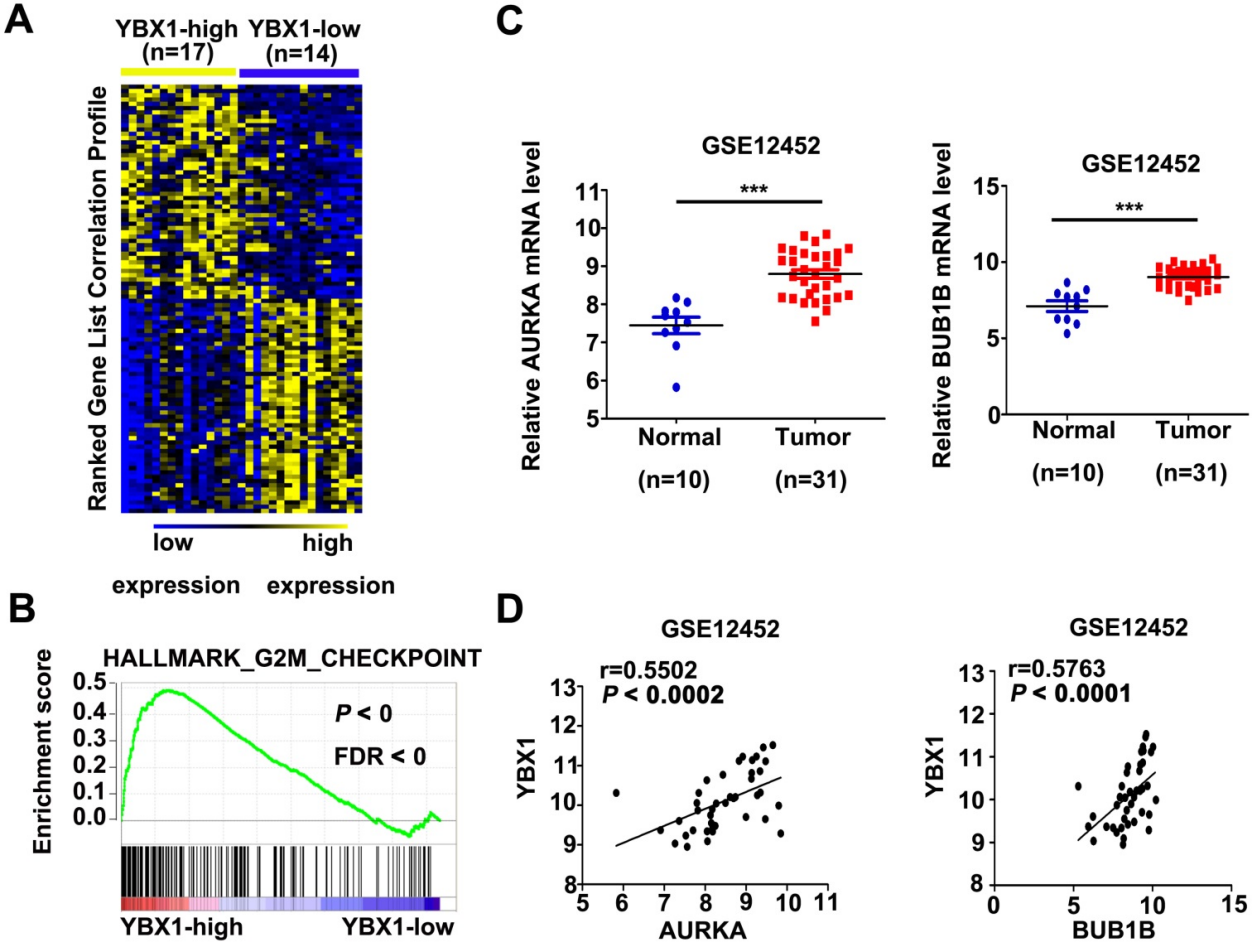
** Correlations between YBX1 and AURKA/BUB1B mRNA levels in NPC.** (A) Representative differentially expressed genes between YBX1^high^ and YBX1^low^ NPC samples were showed as heatmap. Original data were collected from NPC mRNA expression profile GSE12452. (B) GSEA analysis showed that high expression of YBX1 was positively correlated with overexpression of cell cycle G2/M checkpoint modulators. (C) The mRNA levels of AURKA and BUB1B were overexpressed in NPC. (D) The mRNA level of YBX1 was positively correlated with the mRNA levels of AURKA and BUB1B in NPC. ***P < 0.001.

In order to explore the mechanism of YBX1 in NPC, mRNA profiles of NPC samples from GSE12452 were divided into YBX1^high^ (n=17) and YBX1^low^ (n=14) groups. Differentially expressed genes between YBX1^high^ and YBX1^low^ NPC were calculated by NOISeq method 16. The top 50 upregulated or downregulated genes in YBX1^high^ as compared to YBX1^low^ NPC samples were presented in heatmap (Figure 6A). GSEA analysis revealed that high expression of YBX1 positively associates with gene sets related to G2M checkpoint in NPC (Figure 6B). Several canonical G2/M checkpoint regulators, including AURKA and BUB1B, were included in the top 50 upregulated genes in YBX1^high^ NPC samples. The mRNA levels of AURKA and BUB1B were overexpressed in NPC samples and positively correlated with the level of YBX1 (Figure 6C&D).

### YBX1 enhances the translation of AURKA mRNA in NPC cells

**Figure 7:**
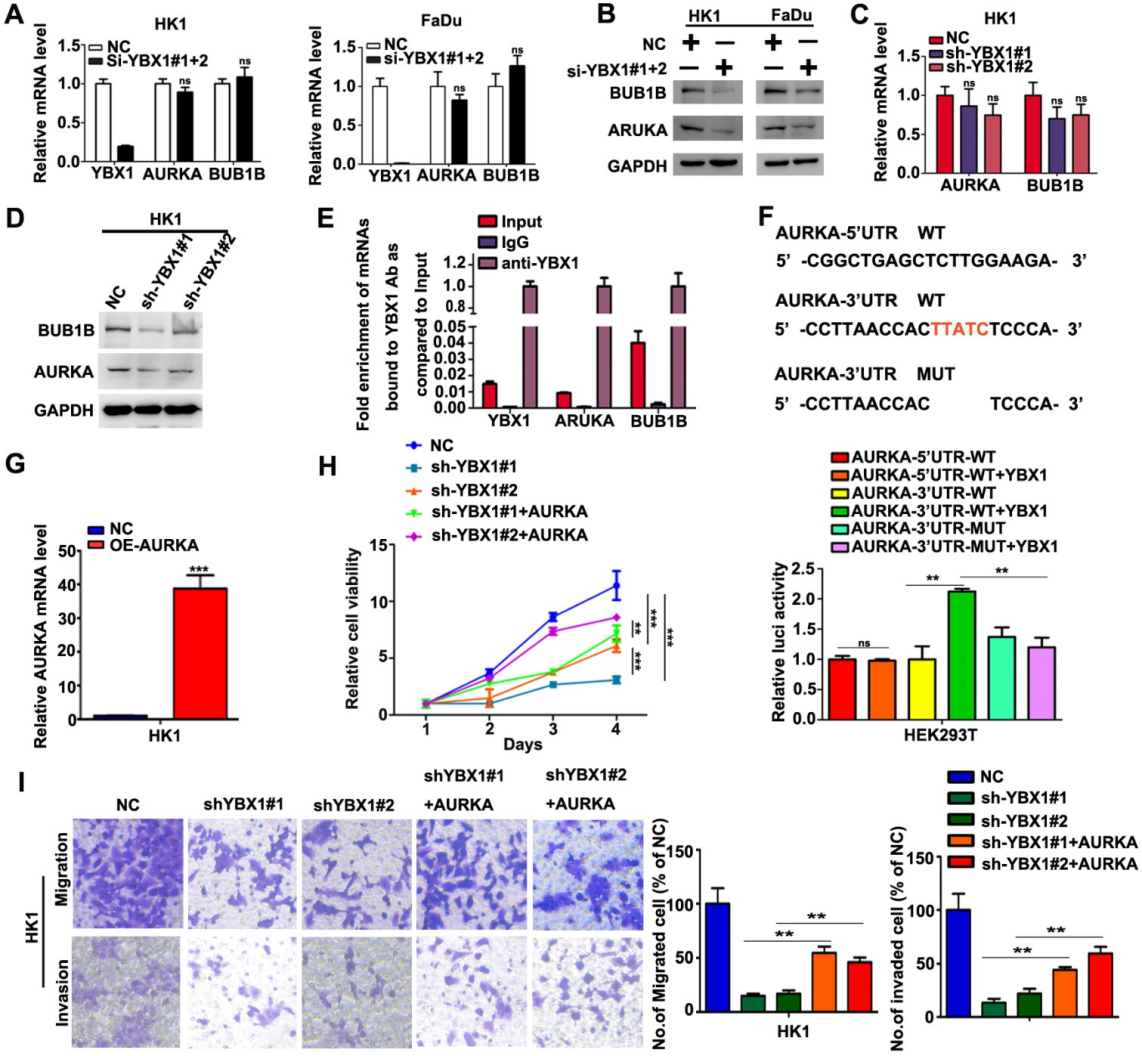
** YBX1 bond AURKA mRNA and enhanced its protein level in NPC cells.** (A) AURKA and BUB1B mRNA levels were determined by qPCR assays. (B) AURKA and BUB1B proteins were measured by western blot assays. (C) AURKA and BUB1B mRNA levels in YBX1-depleted cells were determined by qPCR assays. (D) AURKA and BUB1B protein levels in YBX1-depleted cells were measured by western blot assays. (E) RIP-qPCR assays showed that YBX1 bond to the mRNAs of AURKA and BUB1B. The mRNA of YBX1 itself was used as a positive control for binding assay. (F) Co-transfection of YBX1 plasmids with luciferase reporters inserted with the wild type or mutant untranslated regions which lack of the YBX1 binding site suggested that YBX1 bind to 3'-UTR of AURKA mRNA and enhanced the translation efficiency of AURKA mRNA. (G) AURKA mRNA levels were measured by qPCR assay. (G) Cell proliferation in AURKA plasmids transfected cells were measured by CCK-8 assays. (I) Cell migration and invasiveness in AURKA plasmids transfected cells were measured by transwell assays without or with Matrigel, respectively. **P<0.01. ***P<0.001.

We then asked whether overexpression of YBX1 contribute to elevation of AURKA and BUB1B in NPC cells. As shown in Figure 7A, transient silencing YBX1 did not affect the mRNA levels of AURKA and BUB1B in HK1 and FaDu cells. However, transient silencing YBX1 led to reduction of the protein levels of AURKA and BUB1B in HK1 and FaDu cells (Figure 7B). Similarly, stable silencing YBX1 exerted little effects on the mRNA levels of AURKA and BUB1B in HK1 cells (Figure 7C). However, both AURKA and BUB1B protein levels were reduced in YBX1-silenced HK1 cells (Figure 7D). RIP-qPCR assay demonstrated that YBX1 protein directly bonded to AURKA and BUB1B mRNAs (Figure 7E). Sequence analysis suggested that there were potential YBX1-binding sites (UYAUC) in both 5′-and 3′-untranslated region (UTR) of AURKA mRNA. Then the wild types (WT) of 5′-UTR, 3′-UTR of AURKA mRNA or 3′-UTR mutant lack of YBX1-binding site were sub-cloned into a luciferase reporter vector. Co-transfection of YBX1 plasmids did not affect luciferase activity of WT 5′-UTR-AURKA reporter. However, co-transfection of YBX1 plasmids significantly enhanced the luciferase activity of WT 3′-UTR-AURKA reporter in HEK293 cells, without affecting that of mutant 3′-UTR-AURKA reporter (Figure 7F), indicating an YBX1 specific role involved. Transfection of an AURKA-expressing plasmid in YBX1-silenced HK1 cell led to dramatic increase of AURKA mRNA level (Figure 7G). Functionally, forced expression of AURKA rescued cell proliferation, migration and invasiveness in YBX1-silenced HK1 cell (Figure 7H&I). Thus, our data suggested that YBX1 promotes translation of AURKA mRNA through directly binding to 3′-UTR of AURKA mRNA.

## Discussion

NPC is a kind of malignant tumor with poor prognosis and high frequency of recurrence. In this study, differentially expressed RBPs were firstly identified in NPC. Among these differentially expressed RBPs, YBX1 was overexpressed in NPC and plays an oncogenic role in NPC progression.

YBX1, also known as YB-1, is a multifunctional DNA/RNA binding protein 14, 17. YBX1 belongs to the YBX family which includes YBX1, YBX2 and YBX3 18. YBX1 protein contains three functional domains, including the classical cold shock protein (CSD) domain, A/P domain and a long C-terminal domain with alternating positively and negatively charged amino acids 19. As a RNA-binding protein, YBX1 plays essential roles in multiple aspects of RNA dynamic, including Pre-mRNA splicing, mRNA packaging, translational regulation and exosome miRNAs sorting, etc.^18^. YBX1 acts as an oncoprotein in a variety of human cancers, but its role in NPC remains elusive. We demonstrated that YBX1 mainly distributes in cytoplasm of NPC cells, suggesting YBX1 primarily acts as a RNA binding protein in NPC despite of that it also could translocate into nucleus. *In vitro* and *in vivo* functional assays indicated that overexpression of YBX1 enhances the malignant features of NPC. These data clearly show that YBX1 is an important oncoprotein contributing to the development of NPC.

The previous study reported that silencing YBX1 results in G2/M phase cell-cycle arrest 20, without understanding the underlying detail mechanisms. In the current study, we found that overexpression of YBX1 in NPC is closely correlated with enrichment of mRNAs for G2/M checkpoint regulators in NPC samples. Thus, the current study suggested that dysregulation of RNA binding proteins account for disorder of post-transcriptional processing of key molecules that are critical for cell cycle progression in NPC.

Among these G2/M checkpoint regulators, AURKA and BUB1B were highly expressed in NPC samples and have been recognized to be the critical hub genes in NPC gene regulatory networks 21, 22. We further demonstrated that silencing YBX1 caused reduction of the expression levels of AURKA and BUB1B proteins, without affecting its mRNA expression levels. YBX1 directly bonded to the mRNAs of AURKA and BUB1B. By using a luciferase based reporter system, we further demonstrated that a specific binding motif at the 3'-UTR of AURKA mRNA is primarily responsible for YBX1-mediated upregulation of AURKA protein level in NPC cells. Because YBX1 did not affect the mRNA level of AURKA, it is likely that binding with AURKA by YBX1 would not affect the stability of AURKA mRNA. Thus, upregulation of AURKA protein level by YBX1 is more likely resulted from enhancement of the translation efficiency of AURKA mRNA. This explanation is supported by the early literatures that YBX1 regulates the translation efficiency of mRNAs of the target genes. For example, YBX1 binds to the conserved GC-enriched sequence on the 5'-UTR of SNAIL1 mRNA, activating cap-independent translation, causing epithelial-mesenchymal transition in breast cancer cells 23. YBX1 also improves the translation efficiency of HIF-1α and c-myc to promote cell growth and metastasis 24-26. Therefore, YBX1 translationally controls of cell cycle progression in NPC cells.

## Conclusions

In this study, we established a differentially expressed RBPs profile associated with NPC development. YBX1, an overexpressed RBP in NPC samples, is a critical oncoprotein contributing to progression of NPC through translationally controlling AURKA expression in NPC. This study shed light on the development of novel targeted therapeutic approaches for NPC treatment.

## Abbreviations

RBP: RNA-binding proteins
NPC: nasopharyngeal carcinoma
YBX1: Y box binding protein 1
AURKA: Aurora kinase A
BUB1B: Mitotic checkpoint serine/threonine kinase B
GEO: Gene Expression Omnibus
GSEA: Gene set enrichment analysis
